# A National Undertaking in Difficulties

**Published:** 1909-05-15

**Authors:** 


### Nursing Administration.

### A NATIONAL UNDERTAKING IN DIFFICULTIES.

Queen Victoria's Jubilee Institute for Nurses is an association of which the country is justly proud. Founded by the late Queen, it has always been the object of special interest to the Royal Family, and has enjoyed in no common degree the honorary services of eminent philanthropists and financiers, no less than that of experts in the departments of social life with which it is concerned. It would be difficult to point to a better administered charity, or one which commands more universally the public confidence. Hence the note of depression sounded by the report recently issued over the signature of Lord Goschen is very remarkable, pointing as it does to alterations in conditions under which the work is carried on such as ought not to be ignored.

The financial position of the fund is still matter for grave anxiety. The result of the special appeal made in 1908 wiped out the deficit for that year, and the balance will be sufficient to meet the demands made upon the Institute's resources during the present year. But the treasurer warns subscribers that there will again be a serious deficit in the annual income in 1910. If this were all there would be no need for real anxiety. Many excellent and prosperous societies live from hand to mouth, and the influential character of the council leaves it improbable that funds will be lacking for necessary extensions. The crux of the matter lies in the record of work accomplished. The primary object of the Jubilee Institute is to supply nurses for work among the poor. The methods employed are to procure candidates qualified by three years' training in general hospitals or in Poor-law Infirmaries, and to give them an extra year's training at some centre for district work where Queen's nurses are employed. Formerly the fee paid by the Institute for a year's training was £28, but this fee was last year reduced to £20. The number of nurses trained through the agency of the Jubilee Institute during 1908 was 211, of whom 30 were trained for midwifery, seven in hospitals, and 174 in district work after general training. Including 33 Queen's nurses who rejoined the Institute after having resigned, the number of nurses enrolled during 1908 was 241. This number is very far from adequate to the requirements. There are 829 nursing associations affiliated to the Jubilee Institute and employing or desirous of employing Queen's nurses, but 37 associations severed their connection with the Society chiefly because the Institute was not in a position to supply Queen's nurses for their needs. There is a shortage of suitable candidates willing to accept the benefit of free training offered by the Institute. And there is also a shortage of vacancies in the training centres, although this has been to some extent overcome by recent negotiations. This is not all.

The number of resignations every year about equals the number newly trained. In 1908 211 women were trained, while 203 resigned, the resignations amounting to 13.8 per cent. of the total number of Queen's nurses employed. This is no abnormal year, for in 1907 the figures were much the same. The nursing profession is always in a state of flux, but, even including deaths, the rate of falling off in societies and co-operations ought not to exceed 10 per cent. If the resignations were for the most part from nurses who had borne the burden for many years, it would be comprehensible. But particulars are given of the nurses who resigned, and it appears that only 36 out of the number, or 17.7 per cent., had remained in the service of the Institute as long as six years, while no fewer than 55, or 27 per cent., failed to complete even two years' service after the expensive training given them.

The Institute is face to face, then, with a situation of extreme difficulty. The Chairman of the Council stated, in commencing his report, that "early in 1908 the position became critical. It was a question whether the Institute could follow its hoped-for line of development into a national work, or whether it must be restricted to its existing and comparatively narrow limits." This question the Council is hopeful of having solved by inducing training centres to receive more candidates. But, it will be asked, is not some more fundamental alteration necessary if the work is to lead the way as it ought to do in organising the nursing of the sick poor? Out of the £11,296 expended last year, nearly £5,000 was devoted to giving 211 fully trained nurses experience in district work, preliminary to their becoming Queen's nurses, and out of this number it may be reckoned that about 27 per cent. will continue only a year in the posts which are found for them. Judging by the figures given in the last two reports, by the end of next year there will remain at work only about half the number of those who have been trained at this cost, and in six years' time nearly all will have melted away into other callings. Is the Institute trying to force up the standard higher than the conditions of district work will admit? Are they endeavouring to secure women for these posts by the promise of free training who find subsequently that they can make a better living in other directions? We hope it may prove that other causes are at work than the one we have indicated as possible, for the maintenance of a high standard of training in district work is indeed a matter of national importance. At the same time, facts ought to be faced. The tendency among district nurses is to remain almost as fixtures in the towns and villages where they once settle down, and the reasons for these resignations ought to be investigated by the committee.

There is yet another vital matter to be considered. Is there a certain amount of overlapping going on in connection with the admirable inspection work of the Institute? Inspection and superintendence cost the Society last year £2,714, and we learn from the report that this amount is likely to be considerably increased this year. Might not some of this work be conveniently reorganised by means of co-operation with the County Councils?

